# Dermatoglyphics in hypertension: a review

**DOI:** 10.1186/s40101-015-0065-3

**Published:** 2015-08-12

**Authors:** Buddhika TB Wijerathne, Robert J Meier, Thilini C Agampodi, Suneth B Agampodi

**Affiliations:** Department of Forensic Medicine, Faculty of Medicine and Allied Sciences, Rajarata University of Sri Lanka, Saliyapura, Sri Lanka; Department of Anthropology, Indiana University, Bloomington, IN USA; Department of Community Medicine, Faculty of Medicine and Allied Sciences, Rajarata University of Sri Lanka, Saliyapura, Sri Lanka

**Keywords:** Dermatoglyphics, Hypertension, High blood pressure, Review, Fingerprint, Palm print

## Abstract

Hypertension is a major contributor to the global burden of disease and mortality. A major medical advancement would be a better means to ascertain which persons are at higher risk for becoming hypertensive beforehand. To that end, there have been a number of studies showing that certain dermatoglyphic markers are associated with hypertension. This association could be explained if the risk toward developing hypertension later on in life is somehow connected with fetal development of dermatoglyphics. It would be highly valuable from a clinical standpoint if this conjecture could be substantiated since dermatoglyphic markers could then be used for screening out individuals who might be at an elevated risk of becoming hypertensive. The aim of this review was to search for and appraise available studies that pertain to the association between hypertension and dermatoglyphics.

A systematic literature search conducted using articles from MEDLINE (PubMed), Trip, Cochran, Google scholar, and gray literature until December 2014. Of the 37 relevant publications, 17 were included in the review. The review performed according to the preferred reporting items for systematic reviews and meta-analyses (PRISMA) statement.

This review showed a fairly consistent finding of an increased frequency of whorl patterns along with a higher mean total ridge count in digital dermatoglyphic results in hypertensive samples compared to controls. However, it was imperative to discuss several limitations found in the studies that could make this association as yet unsettled.

## Introduction

Hypertension is a major contributor to the global burden of disease [1]. Furthermore, it is a significant cause of global mortality [2]. It is estimated that hypertension leads to 9.4 million deaths each year globally [1]. As of 2008, worldwide, approximately 40 % of adults aged ≥25 years had been hypertensive [3]. It was estimated to have close to one billion people with hypertension globally in 2000 and predicted a rise to 1.56 billion by 2025 [4]. Hypertension generally is defined as values ≥140 mmHg systolic blood pressure (SBP) and/or ≥90 mmHg diastolic blood pressure (DBP) [5].

Owing to an increase in health burden and an awareness of hypertension among scientists, various biological parameters that are suspected to be related to hypertension are being studied. For instance, there has been a fairly persistent effort to determine if an association of dermatoglyphics with hypertension exists. Dermatoglyphics is the term applied to the scientific study of fingerprint and palmar patterns along with their quantitative measures [6]. Dermatoglyphic features begin developing during early stages of fetal life and are fully formed by the end of the fourth month of intrauterine life, and very importantly, they do not change throughout postnatal life [7–9].

Essential hypertension has been investigated extensively as well as that of juvenile hypertension. Several studies have provided evidence that dermatoglyphic traits are associated with hypertension. Given these results, it has been conjectured that this association could be explained if the risk toward developing hypertension later on in life is somehow connected with the first trimester expression of dermatoglyphic traits. It would be highly valuable from a clinical standpoint if this conjecture could be substantiated since dermatoglyphic markers could then be used for screening out individuals who might be at an elevated risk of becoming hypertensive. The aim of this review was to systematically search for and appraise available studies that pertain to the association between hypertension and dermatoglyphics.

## Methods

The review was performed according to the preferred reporting items for systematic reviews and meta-analyses (PRISMA) statement [10].

### Literature search strategy

The electronic databases of MEDLINE (PubMed), Trip, and Cochrane were searched for articles published before December 2014. Gray literatures were searched using Google scholar and OpenGrey. The reference lists of the studies selected were manually perused. No entries were excluded based on publication date prior to December 2014 or on language. The general search strategy used for PubMed database consisted of MeSH terms. The following search string was employed—“Dermatoglyphics” [Mesh] OR “Palmar prints” [All Fields] AND (“Hypertension” [Mesh] OR “Blood Pressure” [All Fields]). The MeSH term “Dermatoglyphics” included entry terms: Dermatoglyphic; Plantar Prints/Plantar Print/ Print, Plantar/Prints, Plantar/Fingerprints/Fingerprint under its MeSH tree. MeSH terms “Hypertension” included entry terms: Blood Pressure, High/Blood Pressures, High/High Blood Pressure/High Blood Pressures, under its MeSH tree. The same terms were used in Trip, Cochrane, Google scholar, and OpenGrey literature searches.

### Eligibility criteria and study selection

Identified studies were initially filtered with a title search by BTBW based on following criteria.

#### Inclusion criteria

1. Cohort and case control studies evaluated associations between dermatoglyphic variables in hands and hypertension, either essential/juvenile, pre hypertension or elevated blood pressure

#### Exclusion criteria

1. Case reports, case series, cross-sectional studies, editorials, and reviews
2. Control group having diseases that are known to result in dermatoglyphic changes (e.g., diabetes)

The titles and abstracts of retrieved studies were independently reviewed by BTBW and SBA before retrieval of full-text articles for further screening. The full-text article was examined wherever abstracts were unclear. Disagreements were discussed with a third reviewer RJM for final selection of studies to be included in the review.

### Data extraction

From each included study, demographic details, diagnostics or inclusion and exclusion criteria used in both case and control groups, and qualitative and quantitative dermatoglyphic variables have been evaluated and were extracted by one reviewer BTBW and checked by two others SBA and TCA for accuracy. A third reviewer RJM reviewed selected studies to ensure consistency and consensus on judgments.

## Results

**Fig. 1:**
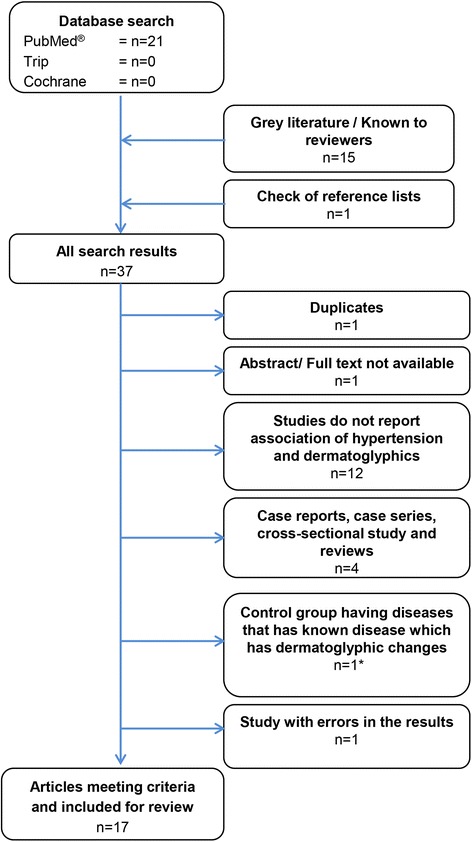
The flow diagram shows the review process and study selection. *(Igbigbi *et al.* 2001 [12])

A search of the scientific databases identified 37 publications with the selected keywords (Fig. 1). Out of this, 20 studies were not suitable for further analysis due to duplication of records (*n* = 1) and do not report association of dermatoglyphics and hypertension (*n* = 12), case series or case reports, or cross-sectional studies or review (*n* = 4). Floris *et al.* [11] had used a control group from another study, and they also mentioned that the possibility of hypertension in their controls; therefore, this study cannot be included in this review. The Igbighi *et al.* [12] study was not included in this review because it was unique in only investigating associations between plantar dermatoglyphics and hypertension, as well as with diabetes. With additional research along this line, perhaps it will be appropriate in the future to assess the value of dermatoglyphics found on soles/toes with respect to associations with hypertension (Fig. 1). Full text or abstract was not available for one article [13], and it was not included in the review. Two studies from an Iranian research group [14, 15] were evaluated, and it could not be determined for certain if they pertained to the same study population. However, since the earlier study [14] contained what appeared to be errors in presenting tabular material, it was decided to only include the latter study [15] in the review.

A total of 17 articles [15–31] met the inclusion and exclusion criteria. Full texts were available only for 14 articles. Only the abstract was available for 3 articles [19, 22, 23].

From selected 17 studies, nine studies describe the association of dermatoglyphics with essential (primary) hypertension. The diagnostic criteria were incorrect in one study [16] which included prehypertension (SBP 120–139 mmHg or DBP 80–89 mmHg [32]), and patients were also classified as primary hypertensives. The significant differences in qualitative or quantitative traits have been observed in all these studies. Main findings are summarized in Table 1. Two studies describe the association of dermatoglyphics with juvenile hypertension. Both these studies showed significant differences in qualitative or quantitative traits, and the findings are summarized in Table 2. Four studies describe dermatoglyphics in hypertension in general, and the findings are summarized in Table 3. Two out of these four studies show significant differences in qualitative or quantitative dermatoglyphic traits with hypertension. Table 4 summarizes the main findings of two studies that assessed dermatoglyphic variables with changes of blood pressure but not in hypertensive patients

**Table 1:** Summary of studies that assessed association of dermatoglyphics with essential (primary) hypertension

Author	Country	Group	Ethnicity	Age	Number ofparticipant	Sex	Selection criteria	Dermatoglyphic findings
Kulkarni SKG *et al.* [16]	India	Case	NR	NR	200	M = 104	BP above 120/80 mmHg. No other secondary diseases causing hypertension	Qualitative traits In hypertensives: females and males have high whorl and low ulnar loop in both hands
						F = 96	Not suffering from any genetic disorder	Quantitative traits In hypertensives: the atd angle lowers in both hands of females and males, and both sexes have high TFRC in both hands
		Control	NR	30–40 years of age (age matched with cases)	200	M = 104	BP below 120/80 mmHg	
						F = 96	No family history of hypertension and not suffering from any genetic illness. First degree relatives clinically screened for genetic disorder. Age matched with cases	
Tafazoli *et al.* [15]	Iran	Cases	NR	Average age = 60 years	40	NR	Patients with essential hypertension	Qualitative traits In hypertensives: much higher frequency of whorls. Significantly larger atd angle on left hand of females
		Control	NR	NR	20	NR	Healthy people. No family history of high blood pressure	
Bulagouda *et al.* [17]	India	Cases	NR	20–50 years of age	100	M = 50	Clinically diagnosed cases of essential hypertension. No diseases causing secondary hypertension. No chromosomal abnormalities	Qualitative traits In hypertensives: the right and left hand showed more arches and radial loops in both sexes, Less ulnar loop in both hands in both sexes, only the right hand of female show more whorls, and presence of Sydney line Quantitative traits In hypertensives: TFRC is significantly lower in males.
						F = 50		
		Control	NR	20–50 years	100	M = 50	Age and sex matched control group	
						F = 50		
Deepa G [18]	India	Cases	NR	NR	100	M = 50	Essential hypertensive patients visiting OPD and IPD	Qualitative traits In hypertensives (males): first and fourth digits showed more whorls and arches and less loops. In hypertensives (females): the digit 1 of females showed more whorls and arches and less loops while digit 2 showed more whorls, loops and less arches. Quantitative traits In hypertensives (for both sexes): decreased a–b ridge counts, decreased adt angle and lower dat value, higher RC in first digit and fifth digit. The lower RC In second digit of left hand and higher ridge count in right hand on the same digit. In hypertensives (males): reduced a–b ridge count, reduced adt angle and more 11 9 7 5’ 13’ main-line formula, increased RC in D1, D3, and D5. In hypertensives (females): Increased “dat” angle, more 11 9 7 5’ 13’ main-line formula D1 and D2 have higher ridge counts
						F = 50		
		Control	NR	NR	100	M = 50	Healthy subjects	
						F = 50	Other selection criteria’s NR. (matched for sex, lifestyle and economic status of cases)	
Kachhave *et al.* [19]^a^	India	Cases	NRA	NRA	60	NRA	Essential hypertensive patients visiting OPD or admitted in medical wards	Qualitative traits In hypertensives: ulnar loop frequency is low. Quantitative trait In hypertensives: TFRC is higher and atd angle showed a significant decrease in both hands
		Control	NRA	NRA	60	NRA	Selection criteria for normal individuals NRA	
Oladipo *et al.* [20]	Nigeria	Cases	Indigenes of Rivers State of Nigeria	35 above	50	M = 26	BP measured to confirm hypertension	Qualitative traits In hypertensives: the whorls showing the highest frequency in most digits of both the right and left hands of males and females. The whorls on the first digit of right hand were strongly associated with essential hypertensive patients. No associations observed for t t^11^ t^111^. Quantitative traits In hypertensives (for both sexes): TRC for each finger is higher. In hypertensives (male): higher atd and lower dat angles of the left hand
						F = 24		
							Secondary causes excluded with assistance of consultant	
		Control	Indigenes of Rivers State of Nigeria	35 above	50	M = 26	Healthy subjects	
						F = 24	Selection criteria’s NR	
Vidya *et al.* [21]	India	Cases	South Indian	40–60	200	M = 100	Essential hypertensive patient attendinginpatient medicine department and OPD. Not having diseases causing secondary hypertension	Qualitative traits In hypertensives (male): less hypothenar pattern in left hand and Simian type 1 pattern less observed. In hypertensives (female): less interdigital area 2 pattern in females (both hands combined) compared with controls. In hypertensives (male): in hypothenar area, left hand had fewer patterns than controls
						F = 100		
		Control	South Indian	Matched control	200	M = 100	Matched for sex, lifestyle, and economic status	
						F = 100		
Kulkarni DU *et al.* [22]^a^	Western Maharashtra India	Cases	Western Maharashtra population	NRA	NRA	NRA	Clinically diagnosed and proved essential hypertensive patients	Quantitative traits In hypertensives: increased TRC and deceased atd angle
		Control	Western Maharashtra population	50 above	100	NRA	Age above 50 years	
Pursnani *et al.* [23]^a^	India	Cases	NRA	NRA	NRA	Both sexes	NRA	Qualitative traits In hypertensives (females): decreased frequency of axial triradius t in the right palm. In hypertensives (males): axial triradius t’ and t” in the right palm In hypertensives (both sexes): absence of axial triradius in both the palms of an individual was found exclusively in hypertensive cases (10 %) and not in controls. Quantitative traits In hypertensives (both sexes): increased TFRC and decreased atd angle
		Control	NRA	NRA	NRA	Both sexes	NRA	

*M* male, *F* female, *RC* ridge count, *NRA* not reported in abstract, *NR* not reported, *TFRC* total finger ridge count, *TRC* total ridge count, *BP* blood pressure, *OPD* outpatient department, *IPD* inpatient department

^a^Only abstract available

**Table 2:** Summary of studies that assessed association of dermatoglyphics with juvenile hypertension

Author	Country	Group	Ethnicity	Age	Number	Sex	Diagnostic criteria	Dermatoglyphic findings
Palyzová *et al.* [24]	Czech Republic	Cases	Inhabitant Prague population	13–27	172	M = 116, F = 56	Elevated BP detected accidently. Scrutinized to rule out secondary causes of Hypertension	Qualitative traits In hypertensives (both sexes): significantly lower ulnar loops and increase whorls. More frequent occurrence of distal triradius (mostly t^1^) and more missing axial triradius Quantitative traits In hypertensives (both sexes): higher TFRC and significantly high mean atd angles In hypertensives (females): lower a–b ridge count observed in right hand
		Control	Inhabitant Prague population	15–65	240	M = 130	Healthy individuals. Not suffering from high BP. No family history of hypertension or its complications	
						F = 110		
Polat MH *et al.* [25]	Istanbul Turkey	Cases		19–35	21	M = 15	Diagnosed patients with hypertension secondary clinical, biochemical, and radiological causes of hypertension excluded	Qualitative traits In hypertensives (females): Significantly lower ulnar loops and higher whorl. In hypertensives (both sexes): Significantly lower occurrence of loops interdigital area in 111 and higher occurrence in H area. Significantly lower axial triradius t occurrence. Ending of palmer A line is more common in position 4 Quantitative traits In hypertensives (males): significantly higher TRC
						F = 6		
		Control			50	M = 25	Healthy controls	
						F = 25		

*M* male, *F* female, *NR* not reported, *BP* blood pressure, *TRC* total ridge count, *TFRC* total finger ridge count

**Table 3:** Summary of studies that assessed association of dermatoglyphics with hypertension

Author	County	Group	Ethnicity	Age	Number	Sex	Diagnostic criteria	Dermatoglyphic findings
Lahiri *et al.* [26]	West Bengal India	Cases	NR	More than 20 years of age	131	NR	Diagnosed as hypertensive and family history of hypertension	Qualitative traits In hypertensives (both sexes): double loop and arch more and whorl, ulnar loop and radial loop are less.
								Quantitative traits
		Control	NR	More than 20 years of age	145	NR	Normal blood pressure (not diagnosed as hypertensives) and absence of family history of hypertension	In hypertensives (both sexes): average ridge counts per finger were high. Corrected atd angles were high
Umana *et al.* [27]	Nigeria	Cases	NR	NR	118	NR	Clinically diagnosed hypertensive patients visiting OPD	Qualitative traits In hypertensives (female): significantly higher loop and slightly higher whorl and low arch patterns in both hands
		Control	NR	NR	126	NR	Normotensive and no family history of hypertension	
Rashad *et al.* [28]	Island of Oahu Hawaii	Cases	American Japanese	NR	Total 742. (the prevalence of hypertension is 9.2)	Males	Hypertension was diagnosed with published criteria by AHA 1960	No significant difference in qualitative traits (finger patterns) or quantitative traits (TRC and ARC)
		Control	American Japanese	NR	people who did not develop hypertension	Males	People who did not develop hypertension	
Reed T [29]	Indiana USA	Case	NR	Mean 63 years (59–70) at third examination of cohort	308 members of twin cohort	Males	“Hypertensive if first, whether subject was on Anti hypertensive drugs or not. Second, 2 physicians’ diagnostic impression related to hypertension. If the above criteria are not met, thirdly, the blood pressure mean”. If patient not on medication or diagnosed by physician as hypertensive, considered hypertensive if SBP ≥ 140 mmHg and DBP ≥ 90 mmHg	Qualitative traits There are no useful relationships between dermatoglyphics and hypertension or strong relationships between the presence of certain dermatoglyphic markers of impaired fetal development. Except subject with high SBP had lower palmar a–b ridge count. Quantitative traits In hypertensives: subjects with high SBP had lower palmar a–b ridge count. Co-twins showed lower ridge counts on the left hand.
		Control	NR		316 members of twin cohort	Males	Normotensive defined as those who attended 2 out of 3 examinations, was not hypertensive or not on antihypertensive at any 3 examinations during 14–18-year study period	

*M* male, *F* female, *NR* not reported, *TRC* total ridge count, *BP* blood pressure, *SBP* systolic blood pressure *DBP* diastolic blood pressure, *ARC* absolute ridge counts, *AMA* American Heart Association

**Table 4:** Studies that assess associations of blood pressure with dermatoglyphic variables

Author	Country	Group	Ethnicity	Age	Number	Sex	Diagnostic criteria	Dermatoglyphic findings
Stevenson *et al.* [30]	Merseyside England	Cases	NR	Born between 1980 and 1981	128	NR	BP measured with automated oscillometric monitor and using a small adult size cuff (Dinamap 1846SX). The last of the 3 readings of systolic and diastolic blood pressure was used in the analysis	No significant differences
		Comparison group	NR	Age matched	128	Sex matched to cases	Age, sex, and school matched comparison group	
Godfrey *et al.* [31]	Lancashire London	Follow-up study	NR	47–56	139	Both male and female	BP measured with automated recorder (Dinamap) while the subjects were seated. Mean values of 2 BP readings (at 5-min interval) were obtained and used in the analysis	Qualitative traits Mean SBP: 8 mmHg higher in 93 men and women with whorl pattern in 1 or more fingers compared with the other 46 who had no whorls. Whorls on the right hand more strongly associated with higher SBP than whorls on the left hand. Mean SBP rose by. 2.2 mmHg for each additional whorl on the right hand. Quantitative traits Mean ARC: SBP rose by 0.88 mmHg for every increase of 10 in ridge count on the right hand and by 0.53 mmHg for every increase of 10 in ridge count on the left hand. Mean SBP: rose by 0.37 mmHg for every increase of 10 in ridge count. People with narrow atd also had higher SBP, prominent for the right hand. Mean SBP rose by 0.49 mmHg for each degree decrease in palmar angle on the right hand [32]

*M* male, *F* female, *NR* not reported, *ARC* absolute ridge count, *TRC* total ridge count, *BP* blood pressure, *SBP* systolic blood pressure, *DBP* diastolic blood pressure, *ARC* absolute ridge counts

### Characteristics of the studied populations

Out of 17 studies, eight were conducted in India [16–19, 21–23, 26], two in the UK [30, 31], two in the USA [28, 29], two in Nigeria [20, 27], one in Czech Republic [24], one in Turkey [25], and one in Iran [15]. However, only five studies reported the ethnic group or population affiliation [20–22, 24, 28]. Population characteristics were uncertain in two studies due to unavailability in the full text and not reported in the abstract [19, 23].

From the 17 studies reviewed, seven studies described the sex of individuals separately [16–18, 20, 21, 24, 25], two studies were done on males [28, 29], and two studies reported data by combining both males and females [23, 31].

Sex of persons was inconclusive in two studies due to unavailability in the full text and not reported in the abstract [19, 22]. Sex was not reported in two studies [26, 30] though in one of those [30], the case and control samples were matched by sex. The sex-wise dermatoglyphic differences were mentioned in another study even though the number of males and females allocated in cases and control groups was not specified [27].

## Discussion

To our knowledge, this is the first attempt to review association of dermatoglyphics to hypertension. Overall, 17 studies fulfilled the inclusion criteria, and among those, nine studies evaluated essential hypertension [15–23], two studies evaluated juvenile hypertension [24, 25], four studies describe dermatoglyphics in hypertension in general [14, 26–29], and two describe association of dermatoglyphics with the changes of blood pressure [30, 31]. Qualitative and quantitative dermatoglyphic variables (digital and palmar) were commonly investigated.

Firstly, with respect to digital dermatoglyphics, there appears to be a fairly consistent finding of an increased frequency of whorl patterns (at least in either sex and at least for some digits) accompanied by an expected reduction in the frequency of loops. Seven studies fell into this category [15–18, 20, 24, 25]. In one study, Lahiri *et al.* [26], double loops were reported to be more frequent in hypertensives but these patterns are usually classified as a subset of whorls. The Umana *et al.* study [27] did not entirely conform to the general trend since it showed a significantly higher loop frequency along with a higher whorl frequency in the female sample of hypertensives and no significant difference in that of males. Of interest, Godfrey *et al.* [31] reported a strong correlation between a rise in whorl frequency with a rise in blood pressure.

To go along with a higher proportion of whorls, there is also a rather consistent expected finding of significantly higher mean values of total finger ridge count (TFRC) in hypertensives as compared with their controls. Whorls generally do have more ridges than loops, and of course, arches are classified as having zero ridge counts. It also would be anticipated that the pattern intensity index (PII) would be elevated due to a relatively higher frequency of whorls, but this variable was not reported on in any of the studies reviewed. A contrary finding with regard to TFRC was made by Bulagouda *et al.* [17] in that the male sample of hypertensives had a significantly lower mean value for TFRC.

Then, in considering palmar dermatoglyphics, a certain clustering of variables stands out both for their significance of difference between hypertensives and controls as well as their interrelatedness. Hence, in general terms, as there were more hypothenar patterns observed in hypertensives, this meant that the axial triradius was more distally positioned, and that led to a broader (larger) atd angle [15, 20, 24, 26], as well more horizontally arranged ridges on the palmar surface. It is important to note that a more narrow (smaller) atd angle was reported also in four studies [19, 22, 23, 31], and this certainly would sound a note of caution toward accepting the significance of the above clustering of variables. Unfortunately, it was not possible to determine exactly what method was employed for measuring the atd angle based on the position of the axial triradius for these studies, a matter that will be discussed below.

The two studies on juvenile hypertension [24, 25] for the most part conformed to the findings in the essential hypertension study in terms of an increased whorl pattern frequency, higher TFRC means, and larger atd angles.

Beyond the generalized tendencies described above, some studies [18, 20] found various and different dermatoglyphic variables to be significant. For example, the presence of a Sidney line was observed in one study, while lower a–b ridge counts were reported in two other studies [18, 24]. Since these variables were either unique or not found across several studies, it seems prudent at this time to afford them less weight with respect to determining whether or not certain dermatoglyphic features can be associated with hypertension. It is possible that future research will help to establish a better grounded connection for some of these variables.

Prior to accepting even the most conservative interpretation that dermatoglyphic development in utero and later expression of hypertension are in some way linked, it is only appropriate to point out certain caveats or limitations in the current evidence as marshaled by the studies that were reviewed.

Obviously, the very first of these considerations is that pertaining to the proper diagnosis of hypertension. Identifying hypertension, both essential and juvenile, is vital in selecting appropriate cases for studying any possible dermatoglyphic associations. It is important that conventional methods be employed in the taking of blood pressure measurements and from them that standard protocols are followed in defining hypertension. However, we found that only a few of the studies reviewed clearly stated that a standard protocol was used [28–31].

Beyond this basic requirement of proper method, it has been shown that there are a number of etiological factors that not only interact with hypertension, but there are certain diseases, such as hypothyroidism [33], that are also likely to affect dermatoglyphic features. Hence, if an appropriate sample selection of hypertensive cases is not made, there is the potential for there being confounding variables. Consequently, it is necessary to exclude secondary causes of hypertension, as well as to clearly define hypertensive cases as being either essential (primary) or of the juvenile form, in order to avoid this confounding effect. For our review, we attempted to sort out those studies that did not clearly indicate whether their cases were essential or juvenile, nor was it apparent in these studies if secondary causes of hypertension due to disease etiology were excluded from the sampling. These studies appear in Table 3.

Then, the next, and perhaps equally fundamental condition for documenting any possible dermatoglyphic/hypertension associations, is that of adequacy of the control samples. Involved here would be both sample size and composition of the control group. Along this line, in nearly all of those studies found in Table 1, there were concerted efforts to collect matched samples, where individuals were aligned by sex and age, and of course those persons making up the control sample would have been free of hypertension and medically diagnosed to be “normal” in this regard. Several of the studies, however, failed to include sufficient sampling strategy information to determine just how appropriate the control samples were.

However, inadequate sample sizes and lack of reporting sample numbers broken down by sex, especially of the control groups, obviously limit the value of many of the studies [15, 20, 22, 23, 25]. It is uncertain whether the controls provided sufficient representation of dermatoglyphic variation found within the population from which affected cases were drawn. Furthermore, fully described ethnic and/or population identification was lacking in most of the studies. Considering that dermatoglyphic “abnormalities” in these studies were only manifested by the degree and significance of difference in frequency distributions, both qualitative and quantitative, between affected cases and controls, it seems imperative that control samples be well-defined and as representative of their historical population source as possible. Of some interest, in testing the notion of controls, it was found that in two fairly large samples (*N*_1_ = 707, *N*_2_ = 662) presumably drawn from the same general population (European descendant residents of the USA), there were significant differences in either one or both sexes in five of the six interval-level variables analyzed [34]. This might well raise the question of whether or not control samples, even when they nominally are drawn from the “same” ethnic group as the hypertensives, actually were completely representative of that group.

Another dimension of dermatoglyphic variability is that observed between males and females. Although this division was not always adhered to in the studies reviewed, it is important to recognize that sex differences in digital and palmar pattern type frequencies as well as in ridge counts and other measures consistently have been found [35]. Without reporting the composition of a sample in terms of numbers of males and females, it is difficult to ascertain whether sexual dimorphism has impacted the nature of the results.

Staying on this matter of dermatoglyphic variability, there is also the well-established observation of differences between the left and right hands or the presence of bimanual asymmetry [36, 37]. This form of bilateral asymmetry when applied to digits can of course deal with individual fingers from the left and right sides or across the summed values for each hand. Bimanual asymmetry has been investigated with regard to digital and palmar pattern type frequencies as well as with ridge count variables.

It is of importance to mention that there is a fairly extensive literature in evolutionary biology that has dealt with asymmetry, particularly in distinguishing the fluctuating form (FA) from directional asymmetry (DA). In biological anthropology, the theoretical basis for this research is that FA historically has been viewed as an indicator of instability during fetal development or as an index of stress level [38, 39]. These studies dealt with FA with respect to nutritional stress as manifested in the lack of bilateral symmetry in the human dentition. However, results have been inconsistent and contradictory in FA research, as evidenced in a very recent study [40] that failed to support the contention that FA of the cranial bones would be present due to prenatal nutritional stress in mice.

This uncertainty thwarts the kind of marker that could be useful in proposing an in utero association between dermatoglyphics and certain adverse health conditions, such as hypertension. Nevertheless, there have been some positive results. For example, a study not reviewed here found that dermatoglyphic FA was significantly higher in patients with schizophrenia [41], and this confirmed the results of two earlier studies. More recently, it was reported that FA variables (including finger ridge counts, a–b ridge counts, and atd angle) were significantly higher in female breast cancer patients [42]. These studies afford some prospect, admittedly rather slim at present, of a clinical application of dermatoglyphic screening. Accordingly, it is hopeful that future investigations into associations between dermatoglyphics and hypertension will incorporate an analysis of bilateral asymmetry into their research protocol.

The next problematic issue to discuss is that of methodology. While many of the studies were careful to describe their methods for the taking of dermatoglyphic impressions (black ink and printing paper being favored), there was less consistency in describing the standard procedures that they used for classifying and analyzing the prints. To be sure, Cummins and Midlo [6] and Penrose [43] did receive proper attribution by some authors, but there were other studies that did not clearly report on this important research step.

Perhaps to focus on one set of connected variables that presented especially troublesome comparability issues, here let us mention the axial triradius, the hypothenar pattern, the alignment of main-line ridges, and the atd angle. Recall that these variables were highlighted generally for being significantly different between hypertensives and controls. What seemed to be lacking is an awareness of the developmental connections among these variables, since in no single study were all of them included in the analysis.

The atd angle might be additionally pointed to for problematic usage on the grounds of printing difficulties; the atd angle can change depending on pressure applied and whether fingers are closely approximated or splayed [44]. The atd angle is also notable for being sex and age-related: the angle tends to be broader in males, and it decreases with age as the palm grows more in length than in breadth [35]. A seemingly unresolved issue regarding the atd angle stems from the variable practice of using the most distally located axial triradius (t”) in its computation if a hypothenar pattern is present, to measure only from the proximal t location, or to use multiple measures from each of the possible locations, t, t’, and t”. Of some relevance, a correction formula has been devised [45] and used in one of the reviewed studies [26] that handles lateral deviation of the axial triradius but does not address the age-relatedness of the atd angle. While complete conformity is not to be expected, it is imperative that the exact method used in determining the atd angle be detailed in the report.

Lastly, and very importantly, in attempting to address the many issues that prompt reservations against accepting the general trends of dermatoglyphic associations with hypertension, there are those studies that do not support these associations [28–30]. Considering the studies in turn, Rashad and Mi [28] stated that hypertensive subjects “were not significantly different in most dermatoglyphic traits from the remaining group” and presented no more detailed results. It is not entirely clear how to assess their negative findings. Reed [29] raised the interesting possibility that unless twins in his samples differed from singletons with regard to in utero developmental conditions, he could find no useful relationships between hypertension and dermatoglyphics. Stevenson, *et al.* [30] provided a thoroughly described and carefully executed study that included the dermatoglyphic variables of digital pattern type frequencies and palmar atd angle as investigated in an index group (defined as very low birth weight (VLBW)) and a comparison group matched for sex, age, and school attended. Sample size for each group (*n* = 128) was adequate, blood pressure readings were meticulously made, and dermatoglyphic analysis appeared quite standard, although it would have helpful to learn more about the Dermaglyph 2.1 program that carried out the analysis of the atd angle. The atd angle is age-sensitive since it is subject to change during the period of growth as noted above [35]. Stevenson *et al.* [30] concluded that they “could find no evidence that higher systolic blood pressure associated with VLBW could be attributed to, or that dermatoglyphic patterns could be markers of, fetal growth” (p. F21). Although this study rightly had matched the index and comparison groups for sex, there might have been additional information if there had been a breakdown by sex.

These three studies showing negative results must be appreciated for their generally high quality and do raise cautions against fully accepting the linkage that was observed between dermatoglyphics and hypertension in the other studies. Clearly, that acceptance awaits future research that possibly may confirm such a relationship.

Then too, the theoretical basis or conceptual framework for explaining dermatoglyphic/hypertension associations is in great need of refinement. For instance, it is one matter to claim that since dermatoglyphics is ultimately shown to be connected with hypertension through appropriate statistical analysis, these traits and that condition are in fact interacting with each other in some manner, or possibly with another agent altogether, during early fetal development. However, it is quite another task to actually discern the precise nature of this connection, beyond a commonly made assertion, that it is very likely to be a combination of hereditary and in utero environmental factors.

One of the studies [30] did indeed raise the issue of fetal development and uterine environment, as connected with subsequent adult risk for disease within the framework of the “Barker” hypothesis. Barker [46] proposed that as a mother’s body experiences nutritional stress, it can set or program the metabolic rate of her developing fetus so that it can adapt to a later adulthood of nutritional insufficiency. However, adverse health consequences await those infants who grow and develop in an undesirable environment of nutritional excess, a situation widely known at present to result in metabolic syndrome. As it turns out, Stevenson *et al.* [30] were unable to support this hypothesis with their dermatoglyphic findings, and negative results also were found by several others who had tested the Barker hypothesis directly utilizing maternal nutritional status.

On the other hand, considerable support for basic features of the hypothesis has appeared under the concepts of “thrifty phenotype” and “developmental programming” [47 – 49]. These concepts greatly advance thinking beyond the outdated notion of nature vs nurture to a level, admittedly more complex, involving interactions between heredity and environment, and very importantly, the increasing relevance of epigenetic factors in altering the phenotypic outcome, particularly during fetal development. It seems quite compelling to view dermatoglyphic markers in light of these proposed mechanisms. Accordingly, it could prove significant to search for evidence that during dermatoglyphic development, a suite of mechanisms connected with metabolic programming and growth parameters interact to bring about potentially diagnostic dermatoglyphic markers of adverse health conditions, such as hypertension in later life. Importantly, mechanisms that were discussed by two of the studies [24, 31] implicated the timing of volar pad development and subsequent regression. Volar pad shape on the fingertips and palms of the fetus during the initial trimester are critical to pattern type formation, that is, whether ridges will form whorls on more elevated, hemispheric pads or form arches on a lower, flatter shaped pad. The two studies cited above proposed that the occurrence of edema in these pads or an increased blood pressure and/or flow to them could have led to a higher frequency of whorl patterns, one of the major findings in hypertensive patients. Obviously, timing of intrauterine events is critical to the expression of dermatoglyphic pattern outcome [50]. Delayed timing might well to lead to different patterns than accelerated development. Hopefully, research along these lines of tracing out fetal developmental mechanisms and in utero conditions will continue to advance our knowledge with respect to associations between dermatoglyphics and hypertension.

## Conclusions

Considering the overall findings in the studies reviewed, hypertensive patients tended to have an elevated frequency of digital whorl patterns that goes along with their having higher average ridge counts than controls. However, at this time, it seems unwarranted to conclude that an intrinsic association between the fetal development of dermatoglyphic features and the adult affliction with hypertension has been satisfactorily demonstrated.

## Abbreviations

BP: blood pressure
DA: directional asymmetry
DBP: diastolic blood pressure
DU: data unavailable
F: female
FA: fluctuating asymmetry
IPD: inpatient department
M: male
NR: not reported
NRA: not reported in abstract
OPD: outpatient department
SBP: systolic blood pressure
TFRC: total finger ridge count
TRC: total ridge count
VLBW: very low birth weight
